# Swallowing and Oral‐Sensorimotor Characteristics in a Sample of Young Hospitalised Children With Severe Acute Malnutrition

**DOI:** 10.1111/jpc.16790

**Published:** 2025-02-08

**Authors:** Casey Jane Eslick, Alta Kritzinger, Marien Alet Graham, Esedra Krüger

**Affiliations:** ^1^ Department of Speech‐Language Pathology and Audiology University of Pretoria Pretoria South Africa; ^2^ Department of Science, Mathematics and Technology Education University of Pretoria Pretoria South Africa

**Keywords:** feeding difficulties, infant and young child feeding (IYCF), oral‐sensorimotor characteristics, severe acute malnutrition (SAM), swallowing

## Abstract

**Aim:**

To describe swallowing and oral‐sensorimotor characteristics in a sample of young hospitalised children with severe acute malnutrition (SAM).

**Methods:**

The Schedule of Oral‐motor Assessment was used to compare oral‐sensorimotor skills of 45 hospitalised patients with SAM (*M* = 15.98 months; SD = 8.03), to pairwise‐matched controls (*M* = 15.96 months; SD = 8.08). Participants were matched for age, gender, TB, HIV‐status, socio‐economic status including maternal education, paternal employment, housing and transport access. Caregivers reported demographic information, feeding practices and behaviours during feeding.

**Results:**

In comparison to controls (*n* = 6; 13.3%), participants with SAM (*n* = 25; 55.6%) presented with significantly more oral‐sensorimotor difficulties (*p* < 0.001), across all consistencies except liquids from the trainer cup and bottle. Difficulties included jaw opening and stabilisation, tongue and lip control for chewing, bolus formation and transport, sustained bite and uncoordinated swallowing. Delayed sitting development significantly correlated with uncoordinated swallowing and head extension on puree and cup drinking, and multiple swallows on puree and semi‐solids. Force‐feeding and slow swallow initiation in semi‐solids significantly correlated. Disruptive feeding behaviours and higher levels of food refusal at the beginning of meals were reported. Clinical signs of aspiration were identified.

**Conclusions:**

Oral‐sensorimotor dysfunction and possible aspiration were observed in 55.6% of participants with SAM. Safe swallowing function for overall health and nutritional recovery is emphasised. Under‐identification of oral‐sensorimotor difficulties, lack of referral to speech‐language therapists and disruption to continuity of care warrants further research.

**Summary**

- What is already known?
  ○ Young children with severe acute malnutrition (SAM) present as weak, hypotonic and lethargic, contributing to motor delays and reduced appetite during in‐patient SAM recovery.
  ○ Mealtimes have been described as disruptive including food refusal in children with SAM.
  ○ Oral‐sensorimotor dysfunction hinders functional feeding abilities and is an associated risk for undernutrition.
  ○ Limited information exists on swallowing and oral‐sensorimotor characteristics of young children with SAM to guide early feeding intervention.
- What this paper adds?
  ○ This paper describes oral‐sensorimotor skills and swallowing characteristics of young children with SAM, highlighting a potential bidirectional relationship between malnutrition and oral‐sensorimotor dysfunction.
  ○ The research group showed significantly more oral‐sensorimotor difficulties than matched controls, across all consistencies except liquids from the trainer cup and bottle.
  ○ Gross motor milestone delay for sitting correlated with uncoordinated swallowing and head extension on puree and cup drinking, and multiple swallows on puree and semi‐solids, emphasising the interplay of nutrition, feeding, and motor development.
  ○ This study highlights that oral‐sensorimotor dysfunction often remains unidentified in young children with SAM, which can delay early intervention for this population.

## 1 Introduction

In Sub‐Saharan Africa, 2.5 million children are affected by severe wasting and severe acute malnutrition (SAM), contributing to delayed cognitive and motor development [1, 2]. Wasting prevalence increased following the COVID‐19 pandemic [1]. Factors within the child and/or environment may contribute to SAM. Established risks are inadequate feeding practices including limited dietary diversity; small quantities, and early introduction of solids [2, 3], which can affect oral‐sensorimotor development [4]. Furthermore, swallowing dysfunction can contribute to SAM [5].

Studies show feeding behaviours vary with nutritional status [6, 7]. Disruptive feeding practices that prolong mealtimes, such as distraction, wandering and playing while eating, were identified in children with SAM [3, 6, 7]. Limited interest in food, low appetite and food refusal, may contribute to force‐feeding [6]. The influence of under‐developed feeding skills on food refusal remains unclear [4].

Oral‐sensorimotor skills include tongue control, lip closure, chewing, respiratory co‐ordination and sensory perception to support the oral and pharyngeal swallowing phases [5]. Physiological changes as a result of SAM including fatigue, hypotonicity and motor delay, may impact oral‐sensorimotor control, swallowing and increase the risk for oropharyngeal dysphagia [2, 5]. Children with SAM and HIV and/or TB show associated respiratory infections that contribute to swallowing difficulties [5, 8].

Emerging evidence on children with SAM, HIV and TB, identified oral‐sensorimotor dysfunction despite intact pharyngeal swallows [9]. Food acceptance, bolus manipulation, chewing and propulsion, were affected across a variety of consistencies, which potentially impacts nutritional recovery, extends hospitalisation and increases costs [5, 9]. Speech‐language therapists (SLTs) intervene in swallowing and oral‐sensorimotor difficulties [5, 10]. Limited literature describes clinical swallowing and oral‐sensorimotor skills in children with SAM to guide intervention. This study aimed to investigate the swallowing and oral‐sensorimotor characteristics in a sample of hospitalised children with SAM.

## 2 Methods

This prospective study compared the research group (RG) identified at three public hospitals in Tshwane, South Africa, with the control group (CG) identified at primary healthcare clinics in the same areas. Facilities serve diverse populations, where 4% of the population lives in poverty [11].

### 2.1 Participants

Children aged 6– 36 months with parent/s willing to participate were purposively recruited, as the age range is a critical period of neuroplasticity and oral‐sensorimotor development [2, 5]. The RG included medically stable hospitalised children, independently diagnosed with SAM according to WHO anthropometric guidelines [12] (Mid‐upper arm circumference [MUAC] ≤ 115 mm, weight‐for‐height *Z*‐score [WHZ] ≤ −3, and/or presence of bilateral pitting oedema). The CG included children with WHZ ≥ –2 and ≤ +2 as determined by trained healthcare practitioners. Seven children were excluded due to presenting with developmental and congenital conditions: Down syndrome and congenital cardiac anomaly (*n* = 2), cystic fibrosis and congenital cardiac anomaly (*n* = 1), cerebral palsy (*n* = 1), bacterial meningitis and seizures (*n* = 2) and an undiagnosed syndrome (*n* = 1). Because of the high prevalence of HIV and TB in young children in South Africa [9, 11, 13], children with these chronic conditions were not excluded. Rather, HIV and TB status were closely matched according to medical records (Table S1). Children in the surgical ward were not eligible for inclusion.

### 2.2 Measures, Materials and Procedures

Anthropometric measurements and nutritional status were classified independently by healthcare practitioners using national guidelines [13] and WHO child growth standards [12, 14]. While anthropometry methods varied across facilities, classifications followed WHO criteria [12, 14]. Acute malnutrition is a deviation from growth standards due to rapid weight loss, wasting and reduced food intake or illness [12, 13]. Chronic malnutrition that reflects prolonged nutritional deficiency, was not differentiated, potentially impacting interpretation of results. Moderate acute malnutrition (WHZ between −2 and −3) [14] was excluded from the study. SAM was diagnosed as WHZ ≤ −3, MUAC ≤ 115 mm, and/or presence of bilateral pitting oedema [12, 14].

To prevent bias, CG participants were identified and pairwise‐matched to RG participants according to key variables. The first available match was selected. RG‐CG pairs were matched for geographic location, age (within 3 months), gender, TB, HIV‐status and self‐reported socio‐economic factors of maternal education, paternal employment, housing, transport access and socio‐economic status classified according to national poverty lines [15]. Categories were matched due to an established association with SAM, early communication skills and feeding development [1, 5, 12]. The same assessments were applied for both groups.

RG participants were assessed once during the hospital admission, involving the caregiver interview and SOMA evaluation. Assessment occurred in the rehabilitative phase of SAM recovery [12], after feeding orally for 2 days or longer [16]. Semi‐structured interviews with caregivers and medical file perusal obtained socio‐demographic information, infants' early developmental and medical histories, feeding practices and behavioural feeding difficulties.

The Schedule for Oral‐motor Assessment (SOMA) is a valid and sensitive measure that guided observation of oral‐sensorimotor skills, functional feeding and swallowing assessment [10, 17, 18]. The SOMA shows positive predictive value compared with instrumental assessments, is suitable for preverbal children and reliably assesses children 6–48 months [17, 19, 20]. Assessment of purees, semi‐solids, solids, crackers and drinking from a bottle, sippy‐cup or open‐cup was completed with adapted food items, as done previously [10]. A qualified SLT explained procedures for each consistency to caregivers who provided food at the SLTs indication. Caregivers were invited to repeat the feed when criteria for observation were not met. Children were allowed to self‐feed if preferred. Refused or unsuitable consistencies were skipped and the remaining were scored [20]. Oral‐sensorimotor behaviours including lip movement, tongue control, jaw stability and swallow coordination were clinically evaluated per consistency based on the SOMA manual criteria [17]. Skills observed were scored ‘yes’, otherwise ‘no’ was recorded [17, 18]. Indicators of potential oropharyngeal dysphagia included coughing, choking, gurgly voice, residue or reduced swallowing coordination, interpreted alongside parent‐reported feeding behaviours such as refusal, wandering during feeding, prolonged feeding or need for compensatory strategies. Oral‐sensorimotor dysfunction was identified when scores fell below cut‐offs in two or more consistencies or when clinical signs of oropharyngeal dysphagia were observed.

Inter‐rater reliability was confirmed by a second SLT simultaneously scoring 15% of the sample, with statistically significant (*p* < 0.05) positive Spearman correlation coefficients for all consistencies. Feeding behaviours and clinical signs of aspiration were observed [5]. Feedback, recommendations and referrals to onsite SLTs were provided as needed.

### 2.3 Ethics

The regional and institutional research committees granted ethical approval (GP_202011_043; HUM009/0320). Informed consent was obtained from caregivers. Participants received intervention at the facility when referral was needed.

### 2.4 Data Analysis

Analyses were conducted using the IBM Statistical Package for Social Sciences, version 25. The SOMA uses binary scoring with cut‐off scores to determine oral‐sensorimotor skills per consistency. Frequency counts and percentages were calculated for each consistency and oral‐sensorimotor skill. Disruptive feeding behaviours and observed clinical signs of aspiration were summarised. Pairwise deletion was used for missing values. Mann–Whitney (MW) tests and two‐proportions *z*‐tests (*z*) were used to determine significant differences, Spearman's correlations and point‐biserial correlations were calculated to identify significant relationships (*p* < 0.05). Inferential statistics including correlations, corresponding *p* values and inter‐rater agreement are reported. The statistical power was 0.822, 0.992, 0.978 for the MW test, the two‐proportions *z*‐test and correlations, respectively.

## 3 Results

### 3.1 Participant Characteristics

Forty‐five RG‐CG pairs (*n* = 90) were included. The age ranges from 6 to 35 months for both groups (RG: *M* = 15.98, SD = 8.03; CG: *M* = 15.96, SD = 8.08). No significant differences were identified between the RG and CG‐age, gender and matched criteria, while all measures of anthropometry showed significant differences (Table S1). All RG participants had previously been discharged from hospital after their birth and re‐admitted prior to inclusion in the study. It was observed that some children were hospitalised due to complicated SAM following assessment at primary healthcare clinics, while others were diagnosed with SAM following hospital admission for illnesses such as HIV and TB. Primary causes for hospitalisation were not recorded due to inconsistencies and variances in hospital records and not emphasising admission diagnoses. Twelve (26.7%) RG participants were severely stunted [14]. One (2.5%) CG participant and fifteen (40.5%) RG participants had delayed sitting (*p* < 0.001).

More CG participants' caregivers were mothers (*n* = 23; 51.1%), whereas RG participants were cared for by mothers (*n* = 14; 31.1%), grandmothers (*n* = 16; 35.6%), extended family (*n* = 11; 24.4%) or neighbours (*n* = 4; 8.9%).

### 3.2 Feeding History

Reported feeding history is summarised in Table 1. Significant disruptive feeding behaviours identified included giving sweets in addition to food (RG: *n* = 24; 53.3%; CG: *n* = 9; 20.0%; *p* = 0.001), and force‐feeding (RG: *n* = 34; 75.6%; CG: *n* = 21; 46.7%; *p* = 0.033). Some CG participants reportedly refuse food at the middle (*n* = 17; 37.8%) or end (*n* = 17; 37.8%), while more RG participants (*n* = 20; 44.4%) refuse food in the beginning (*p* = 0.046) of meals.

**TABLE 1:** Birth and feeding history (*n* = 90).

Feeding history	Research Group *n* = 45		Control group *n* = 45		MW test	*z*‐test
	*n*	%	*n*	%	*p*	*p*
Birth history						
Gestational age (weeks) (*M*, *SD*)	45	37.22 (3.20)	45	38.68 (1.88)	0.038*	
Birth weight (g) (*M*, *SD*)	45	2809.33 (888.32)	45	3180.77 (535.61)	0.035*	
NICU admission	18	52.9	13	44.8		0.521
Respiratory distress syndrome	11	25.0	3	6.7		0.018*
Necrotising enterocolitis	6	13.3	1	2.2		0.049*
Neonatal feeding difficulties	24	53.3	18	40.0		0.205
Breastfeeding (BF)						
Ever breastfed	31	79.5	43	95.6		0.023*
Exclusive BF	36	80.0	38	84.4		0.581
Bottle‐fed	39	86.7	27	60.0		0.004*
Introduced to cup	25	55.6	33	73.3		0.078
Age introduced in months (*M, SD*)		12.36 (5.91)		8.33 (3.28)	0.005*	
Complementary feeding (CF)	23	51.1	35	77.8		0.008*
Age introduced in months (*M, SD*)		6.41 (3.25)		6.36 (2.04)	0.794	
Daily feeds						
Give same CF in a day	26	59.1	11	24.4		0.001*
Children share one plate	11	24.4	2	4.4		0.007*
Caregiver opinion						
Child has big appetite	19	42.2	31	68.9		0.011*
Worried about child's sucking/chewing	15	33.3	9	20.0		0.134
Feeding negatively impacts family	13	28.9	6	13.3		0.071

Abbreviations: BF, breastfeeding; CF, complementary feeding; EBF, exclusive breastfeeding; *M*, mean; MW, Mann–Whitney; NICU, neonatal intensive care unit; SD, standard deviation.

* Significant difference (*p* < 0.05).

Twenty‐four RG participants (53.3%) presented with neonatal feeding difficulty, for which twelve (26.7%) received intervention from SLTs. Notably, there was no SLT involvement since participants' neonatal period and no specific swallowing disorder was identified before data collection.

### 3.3 Oral‐Sensorimotor and Swallowing Characteristics

In comparison to the CG (*n* = 6; 13.3%), the RG (*n* = 25; 55.6%) presented with significantly more oral‐sensorimotor dysfunction in the SOMA (*p* < 0.001), across all consistencies except liquids from the trainer cup and bottle (Table 2). Oral‐sensorimotor difficulties and clinical signs of aspiration observed are summarised (Table 3). Relevant statistical information is in Table S2.

**TABLE 2:** Oral‐sensorimotor and swallowing characteristics per consistency in the SOMA (*n* = 90).

Consistency	Research group *n* = 45			Control group *n* = 45			*z‐*test
	*n*	Frequency	%	*n*	Frequency	%	*p*
Puree	45			45			
Adequate oral‐sensorimotor function		19	42.2		39	86.7	< 0.001*
Oral‐sensorimotor dysfunction		25	55.6		6	13.3	< 0.001*
Refused		1	2.2		0	0.0	0.315
Semi‐solid	45			43			
Adequate oral‐sensorimotor function		23	51.1		33	76.7	0.006*
Oral‐sensorimotor dysfunction		21	47.7		8	18.6	0.006*
Refused		1	2.2		2	4.7	0.557
Solid	44			44			
Adequate oral‐sensorimotor function		9	20.5		27	61.3	< 0.001*
Oral‐sensorimotor dysfunction		26	59.0		12	27.3	< 0.001*
Refused		9	20.5		5	11.4	0.535
Cracker	37			36			
Adequate oral‐sensorimotor function		6	16.3		16	44.4	0.036*
Oral‐sensorimotor dysfunction		20	54.0		16	44.4	0.036*
Refused		11	29.7		4	11.1	0.082
Bottle	33			26			
Adequate oral‐sensorimotor function		24	72.7		16	61.5	0.256
Oral‐sensorimotor dysfunction		2	6.1		0	0.0	0.256
Refused		7	21.2		10	38.5	0.157
Trainer cup	32			31			
Adequate oral‐sensorimotor function		8	25.0		10	32.3	0.413
Oral‐sensorimotor dysfunction		11	34.4		8	25.8	0.413
Refused		13	40.6		13	41.9	1.000
Open cup	42			40			
Adequate oral‐sensorimotor function		21	48.8		33	91.7	0.001*
Oral‐sensorimotor dysfunction		16	37.2		3	8.3	0.001*
Refused		5	14.0		4	1.1	0.694

* Significant difference (*p* < 0.05).

**TABLE 3:** RG oral‐sensorimotor difficulties and signs of aspiration during the SOMA (*n* = 25).

Consistency	Oral‐sensorimotor difficulties		Signs of aspiration
	Oral phase	Pharyngeal phase	
Puree	Poor graded jaw opening, inadequate lip control for food removal, insufficient lip closure, drooling, holding food in the mouth, head extension.	Slow initiation, poor coordination of swallow sequence, tongue protrusion and anterior food loss, multiple swallows, food pocketing.	Wet vocalisation, gagging, coughing.
Semi‐solid	Jaw stabilisation required, associated jaw movements, drooling, holding food in the mouth, reduced tongue control, slow bolus formation.	Slow initiation, tongue protrusion, multiple swallows, food pocketing.	Wet vocalisation, coughing.
Solid	Inadequate lip control for food removal, drooling, holding food in the mouth, head extension, reduced tongue elevation and lateralisation, slow bolus formation.	Slow initiation, poor coordination of swallow sequence, tongue protrusion, multiple swallows, food pocketing.	Facial grimace, wet vocalisation, gagging, coughing.
Cracker	Poor graded jaw opening, small vertical jaw excursions, insufficient lip closure during bite, drooling, reduced tongue control for bolus manipulation, poor chewing, slow bolus formation.	Tongue protrusion, food pocketing.	Wet vocalisation, coughing.
Bottle	Poor graded jaw opening, poor lip seal, holding liquid in the mouth.	Poor coordination of swallow sequence, anterior liquid loss, multiple swallows.	Facial grimace.
Trainer cup	Poor lip seal, small vertical jaw excursions, head extension.	Tongue protrusion, multiple swallows.	Facial grimace, wet vocalisation, coughing, choking.
Open cup	Poor tongue control, small vertical jaw excursions, jaw stabilisation required, head extension.	Anterior liquid loss, multiple swallows.	Facial grimace, wet vocalisation, gagging, coughing, choking.

Delayed sitting milestones significantly correlated with uncoordinated swallow sequence (*r*(76) = −0.373, *p* = 0.001) and head extension (*r*(76) = −0.224, *p* = 0.034) during eating puree consistencies and cup drinking (*r*(65) = −0.278, *p* = 0.025), as well as multiple swallows to clear residue on puree (*r*(76) = −0.292, *p* = 0.010) and semi‐solid consistencies (*r*(73) = −0.242, *p* = 0.039). Reported force‐feeding and slow initiation of the swallowing sequence in semi‐solid consistencies showed significant correlation (*r*(85) = −0.221, *p* = 0.042). Significantly more RG participants required SLT referral for dysphagia (RG: *n* = 24; 53.3%; CG: *n* = 2; 4.4%; *p* < 0.001).

## 4 Discussion

This study investigated the swallowing and oral‐sensorimotor characteristics in a sample of hospitalised children with SAM. Difficulties identified include poor graded jaw opening and stabilisation, food removal, tongue protrusion, sustained bite, poor lip closure and uncoordinated chewing during the oral preparation phase and pocketing with multiple swallows to clear during pharyngeal swallows. Oral‐sensorimotor difficulties identified during oral and pharyngeal phases in the physiological process of swallowing correspond with prior research [9]. Findings revealed significantly more oral‐sensorimotor dysfunction in the children with SAM (*n* = 25; 55.6%). Clinical signs of aspiration indicate risk for oropharyngeal dysphagia, causing or as a result of SAM, which may extend feeding times, increase force‐feeding and prolong nutritional recovery [4]. Findings highlight the impact of SAM on physiological systems of children [4, 5, 12]. SLTs have a role in facilitating safe swallowing and effective feeding for this population [5, 16].

Disruptive feeding behaviours in children (food refusal in the beginning of meals) and their environment (giving sweets, force‐feeding) highlight the complex interplay between feeding practices and malnutrition [3, 6, 7]. Most CG participants were cared for by mothers whereas only one‐third of the RG were cared for (and fed) by their mothers. This difference, despite similar contextual factors, highlights the caregiver as an important environmental consideration for children with SAM [7].

Results align with previous research showing high prevalence of neonatal feeding difficulties and inappropriate feeding practices in children with SAM [2, 3, 9]. The relationship between SAM and oral‐sensorimotor skills for swallowing is possibly bidirectional. Neonatal feeding difficulties may have impaired oral‐sensorimotor development, while SAM weakens swallowing muscles due to wasting [2, 10, 16]. As such, potential swallowing dysfunction limits nutritional intake and contributes to SAM [4, 5, 16]. Investigating the intersection of factors is necessary for targeted interventions to address feeding and oral‐sensorimotor skills for feeding development in children with SAM [3, 5, 9].

Twelve RG participants with oral‐sensorimotor dysfunction received neonatal feeding intervention with SLTs, though did not receive further feeding intervention. This shows a breakdown in healthcare continuity that may relate to feeding difficulties emerging when neonatal oral reflexes diminish [5]. Long‐term patient follow‐up is required as feeding difficulties may persist [5]. Future research should explore continuity of care to determine neonatal healthcare retention strategies. Lack of RG participant referral to SLT services before this study resonates with prior research [9], and emphasises under‐identification and limited teamwork and referrals for this population. A holistic approach with interprofessional collaboration is emphasised.

Significant correlation of delayed sitting with some oral‐sensorimotor difficulties in puree, semi‐solid consistencies and cup drinking emphasises the relation of sitting milestone achievement to feeding development [5]. This finding also highlights that developmental delays can influence and be influenced by nutritional status. Children with SAM often have gross motor delays [2]. Eating purees and solids and cup drinking safely requires upright sitting. The need for postural support and importance of gross motor development for advancement of oral‐sensorimotor skills is highlighted [2, 4, 5].

These findings highlight the complexity of holistic SAM management for health professionals. The matched case–control design enhances validity. While pairwise matching controls some bias, selection, recall and residual confounding biases are acknowledged, particularly as a single researcher performed selection. The cross‐sectional nature limits feeding observations and longitudinal research is recommended to investigate changes in oral‐sensorimotor skills and feeding after SAM recovery. Primary causes for hospital admission are not recorded and the hospital setting introduces bias as factors like appetite, pain and discomfort can influence feeding behaviours [5].

Only clinical assessment was used and children with clinical signs of aspiration were referred without instrumental evaluation. Not differentiating acute and chronic malnutrition, may impact the interpretation of findings. Additionally, variations in anthropometric measurement methods across facilities could introduce inconsistencies in nutritional status classification [12], reducing reliability. Future research should include instrumental measurement of aspiration, and standardise anthropometric measurement methods to enhance health outcomes for children with SAM.

## 5 Conclusion

Children with SAM had significantly more oral‐sensorimotor difficulties and possible aspiration. This study shows under‐identification of oral‐sensorimotor dysfunction with lack of referral to SLTs, delaying early intervention. A holistic approach with continuity of care across a continuum is imperative to address feeding practices and oral‐sensorimotor skills for safe swallowing, improved health and nutritional recovery.

## Author Contributions

C.J.E., A.K., E.K. manuscript writing. C.J.E. data collection. C.J.E., M.A.G. data analysis.

## Ethics Statement

This study was approved by Faculties of Humanities and Health Sciences at the University of Pretoria (HUM009/0320), as well as the Department of Health Tshwane Research Committee (GP_202011_043).

## Consent

Before signing consent, parents of participants were informed that results from this study would be published.

## Conflicts of Interest

The authors declare no conflicts of interest.

## Supporting information


**Table S1.** Supporting Information.
