# Supplementary material for: Swallowing and Oral‐Sensorimotor Characteristics in a Sample of Young Hospitalised Children With Severe Acute Malnutrition
Source: J Paediatr Child Health. 2025 Feb 8;61(5):721–8. doi: 10.1111/jpc.16790 (PMC12053209; doi:10.1111/jpc.16790)
Supplement: Supplementary file 1 — Table S1. Supporting Information. [file JPC-61-721-s001.docx]

**Swallowing and oral-sensorimotor characteristics in a sample of young hospitalised children with severe acute malnutrition**

*Submitted to Journal of Paediatrics and Child Health*

**APPENDIX 1: Supplemental material**

***Participant matching criteria***

**Table S1:** Participant matched criteria (n=90)

| **Characteristic** | **Research Group *n=45*** | | **Control Group *n=45*** | | | ***p*-value^#^** |
| --- | --- | --- | --- | --- | --- | --- |
| **Age in months (M, SD)** | 45 | 15.98 (8.03) | | 45 | 15,96 (8.08) | 0.965 |
| **Gender (%)** |  |  | |  |  |  |
| Female | 20 | 44.4 | | 20 | 44.4 | 1.000 |
| Male | 25 | 55.6 | | 25 | 55.6 | 1.000 |
| **Anthropometry** |  |  | |  |  |  |
| ***Weight in kilograms* (M, SD)** | 45 | 10.21 (2.31) | | 45 | 7.30 (1.74) | <0.001* |
| ***Height in centimeters* (M, SD)** | 45 | 78.20 (8.40) | | 45 | 74.02 (9.68) | 0.029* |
| ***MUAC in centimeters* (M, SD)** | 45 | 14.19 (1.37) | | 45 | 11.52 (1.45) | <0.001* |
| **TB (%)** |  |  | |  |  |  |
| No | 33 | 73.3 | | 38 | 84.4 | 0.114 |
| Yes | 12 | 26.7 | | 7 | 15.6 | 0.114 |
| **HIV (%)** |  |  | |  |  |  |
| Exposed | 27 | 60.0 | | 24 | 53.3 | 0.523 |
| No | 22 | 48.9 | | 23 | 51.1 | 0.833 |
| Yes | 23 | 51.1 | | 22 | 48.9 | 0.833 |
| **Income per person per month in ZAR (M, SD)** | 45 | 920.32 (572.34) | | 45 | 1026.29 (682.94) | 0.295 |
| **Paternal employment (%)** |  |  | |  |  |  |
| No | 13 | 28.9 | | 11 | 24.4 | 0.090 |
| Yes | 16 | 35.6 | | 25 | 55.6 | 0.090 |
| **Housing status (%)** |  |  | |  |  |  |
| Informal dwelling | 15 | 33.3 | | 16 | 35.6 | 0.824 |
| Stay with others | 12 | 26.7 | | 11 | 24.4 | 0.809 |
| Renting | 5 | 11.1 | | 4 | 8.9 | 0.725 |
| Own the property | 8 | 17.8 | | 7 | 15.6 | 0.764 |
| Free housing | 5 | 11.1 | | 7 | 15.6 | 0.535 |
| **Transport access (%)** |  |  | |  |  |  |
| Public transport | 44 | 97.8 | | 44 | 97.8 | 1.000 |
| Own car | 1 | 2.2 | | 1 | 2.2 | 1.000 |
| **Maternal education in years (M, SD)** | 42 | 10.31 (2.66) | | 44 | 11.27 (2.15) | 0.087 |

* significant difference (p<0.05)

^#^ if M and SD reported, the p-value corresponds to the MW test, otherwise, if percentages are reported, the p-value corresponds to the two-proportions z-test
FPL, Food poverty line; HIV, human immunodeficiency virus; *M*, mean; MUAC, mid-upper arm circumference; SD, standard deviation; TB, tuberculosis; %, percentage.

***Oral-sensorimotor and swallowing characteristics***

**Table S2:** Oral-sensorimotor skills and signs of aspiration during the SOMA (n=90)

| **Consistency** | **Oral-sensorimotor characteristics/  Signs of aspiration** | **Research Group *n*=45** | | **Control Group *n*=45** | | | | ***z-*test  *p*-value** |
| --- | --- | --- | --- | --- | --- | --- | --- | --- |
|  |  | *n* | Percentage (%) | *n* | Percentage (%) | | |  |
| **Puree** | **Oral-preparatory phase** |  |  |  | |  |  | |
|  | Graded jaw opening | 19 | 43.2 | 35 | | 77.8 | 0.001* | |
|  | Lower lip – draws in around spoon | 27 | 61.4 | 41 | | 91.1 | 0.001* | |
|  | Upper lip - removes food off spoon | 29 | 65.9 | 41 | | 91.1 | 0.004* | |
|  | Lower lip – assists cleaning | 18 | 40.9 | 33 | | 73.3 | 0.002* | |
|  | Drooling | 15 | 34.1 | 7 | | 15.6 | 0.043* | |
|  | Holding food in the mouth | 27 | 61.4 | 10 | | 22.2 | <0.001* | |
|  | Head extension | 9 | 20.5 | 2 | | 4.4 | 0.022* | |
|  | **Pharyngeal phase** |  |  |  | |  |  | |
|  | Smooth swallow sequence | 22 | 50.0 | 40 | | 88.9 | <0.001* | |
|  | Tongue protrusion | 23 | 52.3 | 17 | | 37.8 | 0.169 | |
|  | Anterior food loss | 17 | 38.6 | 8 | | 17.8 | 0.029* | |
|  | Multiple swallows | 20 | 45.5 | 9 | | 20.0 | 0.010* | |
|  | Food pocketing | 9 | 20.5 | 2 | | 4.4 | 0.022* | |
|  | **Signs of aspiration** |  |  |  | |  |  | |
|  | Facial grimace | 4 | 9.1 | 3 | | 6.7 | 0.671 | |
|  | Wet vocalization | 8 | 18.2 | 2 | | 4.4 | 0.040* | |
|  | Gagging | 6 | 13.6 | 0 | | 0 | 0.010* | |
|  | Coughing | 7 | 15.9 | 2 | | 4.4 | 0.073 | |
| **Semi-solid** | **Oral-preparatory phase** |  |  |  | |  |  | |
|  | Graded jaw opening | 19 | 43.2 | 26 | | 63.4 | 0.062 | |
|  | External jaw stabilisation required | 14 | 31.8 | 1 | | 2.4 | <0.001* | |
|  | Associated jaw movements | 30 | 68.2 | 16 | | 39.0 | 0.007* | |
|  | Drooling | 19 | 43.2 | 9 | | 22.0 | 0.037* | |
|  | Holding food in the mouth | 23 | 52.3 | 12 | | 29.3 | 0.031* | |
|  | Head extension | 13 | 29.5 | 6 | | 14.6 | 0.099 | |
|  | Slow bolus formation | 31 | 70.5 | 16 | | 39.0 | 0.004* | |
|  | **Pharyngeal phase** |  |  |  | |  |  | |
|  | Smooth swallow sequence | 18 | 40.9 | 23 | | 56.1 | 0.161 | |
|  | Tongue protrusion | 27 | 61.4 | 15 | | 36.6 | 0.022* | |
|  | Lip closure during swallow | 32 | 72.7 | 36 | | 87.8 | 0.082 | |
|  | Anterior food loss | 14 | 31.8 | 6 | | 14.6 | 0.062 | |
|  | Multiple swallows | 25 | 56.8 | 8 | | 19.5 | <0.001* | |
|  | Food pocketing | 18 | 40.9 | 3 | | 7.3 | <0.001* | |
|  | **Signs of aspiration** |  |  |  | |  |  | |
|  | Facial grimace | 5 | 11.4 | 2 | | 4.9 | 0.277 | |
|  | Wet vocalization | 6 | 13.6 | 1 | | 2.4 | 0.061 | |
|  | Gagging | 2 | 4.5 | 0 | | 0 | 0.167 | |
|  | Coughing | 10 | 22.7 | 1 | | 2.4 | 0.005* | |
| **Solid** | **Oral-preparatory phase** |  |  |  | |  |  | |
|  | Graded jaw opening | 14 | 40.0 | 23 | | 59.0 | 0.064 | |
|  | Lower lip – draws in around spoon | 19 | 54.3 | 33 | | 84.6 | 0.004* | |
|  | Upper lip - removes food off spoon | 23 | 65.7 | 30 | | 76.9 | 0.286 | |
|  | Drooling | 15 | 42.9 | 8 | | 20.5 | 0.038* | |
|  | Holding food in the mouth | 22 | 62.9 | 9 | | 23.1 | 0.001* | |
|  | Head extension | 15 | 42.9 | 7 | | 17.9 | 0.019* | |
|  | Slow bolus formation | 27 | 79.4 | 13 | | 33.3 | <0.001* | |
|  | **Pharyngeal phase** |  |  |  | |  |  | |
|  | Smooth swallow sequence | 9 | 25.7 | 20 | | 51.3 | 0.024* | |
|  | Tongue protrusion | 13 | 37.1 | 26 | | 66.7 | 0.011* | |
|  | Anterior food loss | 14 | 40.0 | 10 | | 25.6 | 0.188 | |
|  | Multiple swallows | 27 | 77.1 | 16 | | 41.0 | 0.002* | |
|  | Food pocketing | 15 | 44.1 | 5 | | 12.8 | 0.003* | |
|  | **Signs of aspiration** |  |  |  | |  |  | |
|  | Facial grimace | 10 | 28.6 | 1 | | 2.6 | 0.002* | |
|  | Wet vocalization | 6 | 17.1 | 1 | | 2.6 | 0.032* | |
|  | Gagging | 6 | 17.1 | 0 | | 0 | 0.007* | |
|  | Coughing | 9 | 25.7 | 1 | | 2.6 | 0.004* | |
| **Cracker** | **Oral-preparatory phase** |  |  |  | |  |  | |
|  | Graded jaw opening | 8 | 30.8 | 20 | | 62.5 | 0.016* | |
|  | Small vertical excursions | 11 | 42.3 | 22 | | 68.8 | 0.043* | |
|  | External jaw stabilisation required | 9 | 34.6 | 6 | | 18.8 | 0.170 | |
|  | Lip closure during bite | 15 | 57.7 | 28 | | 87.5 | 0.010* | |
|  | Sustained bite | 6 | 23.1 | 15 | | 46.9 | 0.061 | |
|  | Drooling | 17 | 65.4 | 13 | | 40.6 | 0.061 | |
|  | Holding food in the mouth | 20 | 80.0 | 20 | | 62.5 | 0.152 | |
|  | Head extension | 14 | 53.8 | 17 | | 53.1 | 0.315 | |
|  | Slow bolus formation | 20 | 90.9 | 20 | | 71.4 | 0.087 | |
|  | **Pharyngeal phase** |  |  |  | |  |  | |
|  | Tongue protrusion | 13 | 50.0 | 9 | | 28.1 | 0.088 | |
|  | Anterior food loss | 16 | 61.5 | 20 | | 62.5 | 0.940 | |
|  | Multiple swallows | 20 | 90.9 | 20 | | 71.4 | 0.087 | |
|  | Food pocketing | 15 | 68.2 | 6 | | 21.4 | 0.001* | |
|  | **Signs of aspiration** |  |  |  | |  |  | |
|  | Facial grimace | 2 | 8.3 | 2 | | 6.5 | 0.790 | |
|  | Wet vocalization | 4 | 16.7 | 1 | | 3.2 | 0.086 | |
|  | Gagging | 3 | 11.5 | 0 | | 0 | <0.001* | |
|  | Coughing | 12 | 50.0 | 3 | | 9.7 | 0.001* | |
| **Bottle** | **Oral-preparatory phase** |  |  |  | |  |  | |
|  | Anticipatory mouth opening | 25 | 96.2 | 16 | | 100 | 0.427 | |
|  | Upper lip – seals tightly | 22 | 84.6 | 15 | | 93.8 | 0.375 | |
|  | Lower lip – seals tightly | 14 | 53.8 | 3 | | 18.8 | 0.024* | |
|  | Small vertical excursions | 22 | 84.6 | 16 | | 100 | 0.099 | |
|  | Holding liquid in the mouth | 7 | 26.9 | 1 | | 5.9 | 0.083 | |
|  | Head extension | 2 | 7.7 | 1 | | 5.9 | 0.820 | |
|  | **Pharyngeal phase** |  |  |  | |  |  | |
|  | Smooth swallow sequence | 13 | 50.0 | 9 | | 56.3 | 0.694 | |
|  | Lip closure during swallow | 21 | 80.8 | 15 | | 93.8 | 0.243 | |
|  | Anterior liquid loss | 4 | 15.4 | 0 | | 0 | 0.089 | |
|  | Multiple swallows | 8 | 30.8 | 0 | | 0 | 0.011* | |
|  | **Signs of aspiration** |  |  |  | |  |  | |
|  | Facial grimace | 1 | 3.8 | 0 | | 0 | 0.413 | |
|  | Wet vocalization | 1 | 3.8 | 0 | | 0 | 0.413 | |
|  | Coughing | 1 | 3.8 | 0 | | 0 | 0.413 | |
| **Trainer cup** | **Oral-preparatory phase** |  |  |  | |  |  | |
|  | Small vertical excursions | 16 | 84.2 | 12 | | 66.7 | 0.214 | |
|  | External jaw stabilisation required | 7 | 36.8 | 4 | | 22.2 | 0.331 | |
|  | Holding liquid in the mouth | 8 | 42.1 | 11 | | 61.1 | 0.248 | |
|  | Head extension | 12 | 63.2 | 9 | | 50.0 | 0.419 | |
|  | **Pharyngeal phase** |  |  |  | |  |  | |
|  | Tongue protrusion | 11 | 57.9 | 7 | | 38.9 | 0.248 | |
|  | Anterior liquid loss | 7 | 36.8 | 9 | | 50.0 | 0.514 | |
|  | Multiple swallows | 13 | 68.4 | 10 | | 55.6 | 0.898 | |
|  | **Signs of aspiration** |  |  |  | |  |  | |
|  | Facial grimace | 4 | 21.1 | 3 | | 16.7 | 0.734 | |
|  | Wet vocalization | 5 | 26.3 | 2 | | 11.1 | 0.238 | |
|  | Gagging | 2 | 10.5 | 6 | | 33.3 | 0.092 | |
|  | Coughing | 9 | 47.4 | 4 | | 22.2 | 0.109 | |
|  | Choking | 8 | 42.1 | 1 | | 5.6 | 0.010* | |
| **Open cup** | **Oral-preparatory phase** |  |  |  | |  |  | |
|  | Small vertical excursions | 16 | 43.2 | 24 | | 66.7 | 0.044* | |
|  | Holding liquid in the mouth | 19 | 51.4 | 13 | | 36.1 | 0.190 | |
|  | Head extension | 19 | 51.4 | 10 | | 27.8 | 0.040* | |
|  | **Pharyngeal phase** |  |  |  | |  |  | |
|  | Tongue protrusion | 24 | 64.9 | 17 | | 47.2 | 0.129 | |
|  | Anterior liquid loss | 32 | 86.5 | 23 | | 63.9 | 0.025* | |
|  | Multiple swallows | 26 | 70.3 | 14 | | 38.9 | 0.007* | |
|  | **Signs of aspiration** |  |  |  | |  |  | |
|  | Facial grimace | 11 | 29.7 | 1 | | 2.8 | 0.002* | |
|  | Wet vocalization | 14 | 37.8 | 4 | | 11.1 | 0.008* | |
|  | Gagging | 11 | 29.7 | 3 | | 8.3 | 0.020* | |
|  | Coughing | 16 | 43.2 | 7 | | 19.4 | 0.029* | |
|  | Choking | 14 | 37.8 | 3 | | 8.3 | 0.003* | |

* significant difference (p<0.05)
%, percentage.
